# A Mini-Review of Reactive Oxygen Species in Urological Cancer: Correlation with NADPH Oxidases, Angiogenesis, and Apoptosis

**DOI:** 10.3390/ijms18102214

**Published:** 2017-10-22

**Authors:** Yasuyoshi Miyata, Tomohiro Matsuo, Yuji Sagara, Kojiro Ohba, Kaname Ohyama, Hideki Sakai

**Affiliations:** 1 Department of Urology, Nagasaki University Graduate School of Biomedical Sciences, 1-7-1 Sakamoto, Nagasaki 852-8501, Japan; tomo1228@nagasaki-u.ac.jp (T.M.); gaasara3@gmail.com (Y.S.); ohba-k@nagasaki-u.ac.jp (K.O.); hsakai@nagasaki-u.ac.jp (H.S.); 2 Department of Pharmaceutical Science, Nagasaki University Graduate School of Biomedical Sciences, 1-7-1 Sakamoto, Nagasaki 852-8501, Japan; k-ohyama@nagasaki-u.ac.jp

**Keywords:** reactive oxygen species, NADPH oxidases, angiogenesis, apoptosis, urological cancers

## Abstract

Oxidative stress refers to elevated reactive oxygen species (ROS) levels, and NADPH oxidases (NOXs), which are one of the most important sources of ROS. Oxidative stress plays important roles in the etiologies, pathological mechanisms, and treatment strategies of vascular diseases. Additionally, oxidative stress affects mechanisms of carcinogenesis, tumor growth, and prognosis in malignancies. Nearly all solid tumors show stimulation of neo-vascularity, termed angiogenesis, which is closely associated with malignant aggressiveness. Thus, cancers can be seen as a type of vascular disease. Oxidative stress-induced functions are regulated by complex endogenous mechanisms and exogenous factors, such as medication and diet. Although understanding these regulatory mechanisms is important for improving the prognosis of urothelial cancer, it is not sufficient, because there are controversial and conflicting opinions. Therefore, we believe that this knowledge is essential to discuss observations and treatment strategies in urothelial cancer. In this review, we describe the relationships between members of the NOX family and tumorigenesis, tumor growth, and pathological mechanisms in urological cancers including prostate cancer, renal cell carcinoma, and urothelial cancer. In addition, we introduce natural compounds and chemical agents that are associated with ROS-induced angiogenesis or apoptosis.

## 1. Introduction

**Figure 1:**
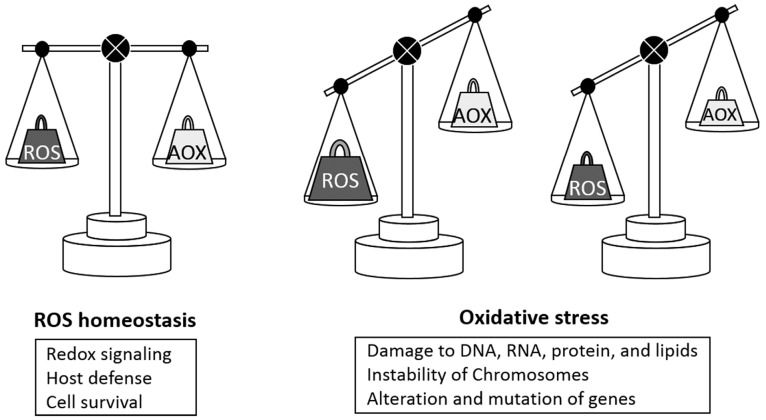
Oxidative stress is defined as an imbalance between the cellular production of reactive oxygen species (ROS) and antioxidants (AOX). Under natural conditions, the production of pro- and antioxidants is in a stable equilibrium. Excessive ROS production or depletion of antioxidants can cause oxidative stress, which may damage cellular lipids, proteins, and DNA, with the latter leading to chromosomal instability and gene mutations.

Oxidative stress is defined as the imbalance between the production of pro-oxidants, such as free radicals and reactive metabolites, and their elimination by protective mechanisms, referred to as antioxidants (Figure 1) [1]. The oxide balance is maintained by an endogenous enzymatic mechanism, but it is also affected by exogenous factors, such as lifestyle, medications, and diet [2,3]. There is a general agreement that oxidative stress is closely associated with various complex physiological and pathological mechanisms [3,4]. In addition, there is growing support for the concept that oxidative stress plays important roles in carcinogenesis, malignant behavior, and prognosis, in various types of cancer [4,5]. Thus, understanding the pathological roles and regulatory mechanisms of oxidative stress in malignant cells and tissues is essential to discuss the observations and treatment strategies in patients with malignancies.

Oxidative stress refers to elevated intracellular levels of reactive oxygen species (ROS). ROS are a heterogeneous group of highly reactive ions and molecules derived from molecular oxygen (O_2_), including superoxide anions, hydroxyl radicals, hydrogen peroxide, HOCl, and singlet oxygen [6]. Although ROS were initially believed to be toxic and associated with various pathological mechanisms, subsequent studies have shown that ROS also play crucial roles in physiological processes [7]. In fact, ROS can act as an antioxidant system to maintain redox homeostasis, even though they are recognized as strong pro-oxidants themselves [8]. Thus, various evidence supports the fact that the biological roles of ROS are complex and paradoxical [7,9]. The degree of ROS production is speculated to be the determinant factor in such dual roles. In short, a low or moderate increase in ROS promotes cell proliferation, apoptosis, angiogenesis, and migration in physiological conditions, and ROS act to maintain cellular homeostasis [10]. In contrast, excessive production of ROS can cause oxidative stress, which may damage cellular lipids, proteins, and DNA [6,11]. Thus, balancing the ROS level is important for regulating cell proliferation, apoptosis, migration, and angiogenesis, and under normal conditions, the production and elimination of ROS are tightly controlled through the help of ROS scavengers (endogenous antioxidants) of an enzymatic (e.g., catalase, glutathione peroxidase system) or a non-enzymatic (e.g., ascorbic acid, lipoic acid, α-tocopherol) nature.

As mentioned above, excessive ROS production causes DNA damage and promotes the activities of oncogenes and/or inhibits tumor-suppressor genes. For example, H_2_O_2_ has been shown to stimulate an activating mutation of the proto-oncogene, c-Ha-ras-1, whereas it inhibits the function of the tumor-suppressor gene, p53 [12]. In addition to such genetic changes, ROS are known to stimulate carcinogenesis via various epigenetic alterations. The methylation of tumor-suppressor genes is the most representative epigenetic alteration in oxidative stress-induced carcinogenesis. For example, H_2_O_2_ has been reported to hypermethylate tumor-suppressor genes, such as retinoblastoma, Von Hippel–Lindau, and breast cancer 1 [13,14]. Furthermore, the activities of various proteins and signaling molecules, such as mitogen-activated protein kinase (MAPK) and extracellular regulated kinase (Erk)1/2 (associated with cell proliferation [15,16]), nuclear factor κB (NFκB) (involved in cell proliferation and the cell cycle [17]), and 3-phosphoinositide-dependent kinase (PDK)-1 (involved in cell proliferation and apoptosis [16]), are affected by ROS. Phosphoinositide 3-kinase (PI3K)/Akt (protein kinase B) signaling, implicated in cancer cell proliferation, is also modulated by ROS [18]. Interestingly, ROS can suppress the activity of phosphatase and tensin homolog (PTEN), recognized as inhibitor of PI3K/Akt signaling [19]. PI3K/Akt signaling is also associated with various malignant behaviors, including apoptosis, angiogenesis, and chemotherapy resistance [18]. Thus, ROS can regulate the activities of cancer-related pathways, via direct and indirect mechanisms.

The mitochondrial electron transport chain is suggested to be the major endogenous source of ROS [20]. Other important sources of ROS are enzymes, such as NADPH oxidase (NOX), xanthine oxidase (XO), and lipoxygenase (LOX) [21]. Among these, special attention has been paid to NOX because it is the best-known source of cellular ROS and because of its cross-talk with mitochondria [22,23]. Furthermore, increased ROS lead to the upregulation of cyclooxygenase (COX)-2 activity [24]. ROS also activate matrix metalloproteinases (MMPs), which can degrade basement membranes and the extracellular matrix [25]. Interestingly, tissue inhibitors of metalloproteinases (TIMPs), endogenous inhibitors of MMPs, are downregulated by ROS [26]. In addition to the MMP/TIMP system, urokinase-type plasminogen activator (uPA) is well known to degrade extracellular compounds, and ROS have been reported to induce uPA expression via regulation of MAPK [15]. Furthermore, ROS directly regulate the activity of β-actin, a regulator of cell adhesion [27]. Previous reports have shown that such ROS-related molecules play crucial roles in carcinogenesis and malignant aggressiveness, via the regulation of cell differentiation, proliferation, invasion, and angiogenesis in several types of cancer [11,16,21,28]. Based on these facts, ROS are speculated to be closely associated with pathological mechanisms as well as with outcomes in cancer patients. In fact, elevated oxidative signaling because of excessive production of ROS has been reported to be associated with carcinogenesis and malignant behavior [28,29,30,31], and cancer cells have higher ROS levels than their normal counterparts [32].

Thus, information on ROS-related molecules and pathways is important to understand the pathological mechanisms and to discuss the treatment strategies in several types of malignancies, including urological cancers. Several investigators are of the opinion that ROS are crucial for cell survival, angiogenesis, invasion, and extravasation [33,34]. Among these biological phenomena, angiogenesis plays crucial roles in cell survival, via the supply of oxygen and nutrients, and cell dissemination in solid tumors. In other words, solid malignant tumors are regarded as “vascular diseases”. In fact, increased levels of ROS have been reported in several cancer cells, and play important roles in tumor development, cell survival, and progression [34,35,36]. Therefore, in this review, we present a simplified outline of the proposed mechanisms of oxidative stress under normal and pathological conditions, including malignancies. Further, we summarize the pathological significance of NOXs in urological cancers, such as prostate cancer, renal cell carcinoma (RCC), and urothelial cancer. Finally, we show the relationships between ROS and apoptosis in pathological conditions, including malignancies, and natural products and chemical agents that influence ROS-related apoptosis in urological cancers are introduced.

## 2. NOX and Urological Cancer

### 2.1. Pathological Significance of NOX1–5 in Malignancies

NOX was first described in the context of leukemia. The NOX family comprises seven isoforms: NOX 1–5 and dual oxidases (DUOX) 1 and 2. NOX 1–5 generate superoxide anions, whereas DUOX1 and 2 generate H_2_O_2_ [37]. As mentioned above, the NOX family is one of the major sources of ROS and one of the key regulators in ROS-related mechanisms expressed in various epithelial cells. In regard to stimuli of mammalian NOXs, chemicals, inflammatory factors, and changed cellular environments are the best known [38,39]. NOX-mediated ROS are reported to stimulate various pro-oncogenes, such as Src and Ras, and to inhibit tumor suppressors, such as p53 and PTEN [40]. In addition, NOXs and NOX-mediated ROS can mediate cellular transformation and genetic programming related to cell growth [40,41,42]. Furthermore, malignant cells generate NOX-dependent extracellular superoxide anions, which contribute to the control of cell proliferation and intracellular ROS/reactive nitrogen species-dependent signaling pathways that cause apoptosis [43,44]. Consequently, during tumor progression, tumor cells establish resistance towards intracellular signaling, though the expression of membrane-associated catalase [45]. Thus, information about the pathological significance of NOXs is essential for understanding the relationship between oxidative stress and tumorigenesis, cell survival and death, cell dissemination, and the prognosis of malignancies.

NOX1 is most highly expressed in the colon epithelium and to a lesser extent in endothelial cells, vascular smooth muscle, and the prostate [41,46,47]. NOX1 has been reported to be closely associated with malignant potential, apoptosis, and angiogenesis, under various conditions [47,48]. The overexpression of NOX1 in NIH3T3 fibroblasts has induced malignant transformation, rendering them slightly tumorigenic in athymic mice [41]. Because of these facts, the function of NOX1 in human cancer has been focused on among the NOX family members [49]. In fact, NOX1 is associated with cell proliferation in colon cancer [50] and carcinogenesis in skin cancer [51].

NOX2 was first described in phagocytic leukocytes and recognized as a mediator of inflammation. NOX2 has also been widely studied in humans. Similar to NOX1, NOX2 is suggested to play important roles in carcinogenesis. Actually, NOX1- and NOX2-induced ROS production is a cause of DNA damage by acidic bile reflux in esophageal cells [52]. From this finding, it has been suggested that inhibition of ROS, induced by reflux, could be a useful strategy for preventing DNA damage and decreasing the risk of tumorigenic transformation caused by gastroesophageal reflux disease, which is the greatest risk factor for esophageal adenocarcinoma. Investigators have also reported pathological roles of NOX2 in malignancies. For example, NOX2-derived ROS facilitate the metastasis of melanoma cells, by downmodulation of NK-cell function [53], immunosuppression by chronic myelomonocytic leukemia (which depends on NOX2 [54]), and cell proliferation—which is associated with NOX2 in gastric cancer [55].

NOX3 is expressed in the inner ear [56]. Increased NOX3 protein expression has been detected after heavy ion irradiation, which also induces other NOXs (NOX 1, 2, 4, and 5) [57]. In non-small cell lung cancer, NOX3 has been linked to tumor growth [58]. The mechanism of NOX3 regulation is less well known than those of other isoforms.

NOX4 is highly expressed in the kidneys [59]. Various growth factors and their receptors, such as insulin-like growth factor (IGF)-1, transforming growth factor β (TGF-β), and toll-like receptor 4 (TLR4), have been reported to stimulate NOX4 activity [60,61,62]. Interestingly, stimulation of the IGF-1 receptor was positively associated with cell survival via induction of NOX4-generated ROS in a variety of cancers [60]. Furthermore, NOX4 is associated with tumor growth and the prevention of apoptosis in the presence of growth factors in several malignancies [63,64]. In addition, NOX4 stimulates angiogenesis through the upregulation of vascular endothelial growth factor (VEGF)-A and hypoxia-inducible factor (HIF)-1α in a variety of cancers [65], and siRNA-mediated knockdown of NOX4 inhibited VEGF-induced endothelial cell migration and proliferation [66]. In a mouse model, NOX4 expression was upregulated in new capillaries in brain ischemia-induced angiogenesis [67]. Thus, NOX4 is suggested to play crucial roles in aggressive malignancy.

NOX5 is detected in various normal tissues, including the fetal tissues, as well as in the adult spleen and uterus [59]. Increased mRNA expression of NOX5 has been detected in cell lines and tumor tissues of various malignancies, including melanoma and breast cancer, but not in colorectal, hepatic, and ovarian cancer and Ewing’s sarcoma [68]. In contrast, NOX5 mRNA expression in testicular tumor tissues was significantly lower than that in the adjacent normal ones [68]. On the other hand, NOX5 is reportedly associated with pathological significance in esophageal carcinoma [69], and plays important roles in angiogenesis via the stimulation of endothelial cells and vascular muscle cells in malignancies [70]. Thus, the pathological functions of NOX5 in malignancies have widely been investigated in both in vivo and in vitro studies.

### 2.2. Pathological Significance of DUOX1 and 2 in Malignancies

In contrast to the quite well elucidated roles of NOXs, the functions of the two DUOX isoforms remain unclear. DUOX1 expression was not detected in lung cancer cells [71]. In addition, the DUOX1 mRNA expression level in hepatocellular carcinoma tissues is lower than that in non-cancerous tissues [72]. Similar results have been found in various other cancers, including esophagus cancer, lung cancer, and thyroid cancer [73]. Thus, DUOX1 expression is suppressed in various malignancies. In contrast, in one report, the reintroduction of functional DUOX1 into lung cancer cell lines increased cell migration and wound repair, without affecting cell growth [71]. In addition, DUOX1 has been identified as a risk factor for the prognosis of hepatocellular carcinoma patients after surgery [74].

DUOX2 mRNA and protein are reportedly expressed in several malignancies, including lung, breast, colorectal, gastric, and pancreatic cancers [75,76]. In contrast, lung cancer cells show losses of DUOX2 expression [71]. In another report, no significant differences in DUOX2 expression were found in carcinomas originating from the breast, lung, skin, stomach, or thyroid [73]. Thus, there is no general agreement on the pathological significance of DUOXs in malignant tumors.

## 3. Pathological Significance of the NOX Family in Urological Cancers

### 3.1. NOX 1–5 in Prostate Cancer

In an in vivo study, the overexpression of NOX1 mRNA was not detected in three prostate cancer cell lines (DU145, LNCaP, and PC-3) [77]. In addition, DU145 cells do not express NOX1 mRNA [78]. On the other hand, one study reported that NOX1 m-RNA is expressed at a low level in DU145 cells and at higher levels in LNCaP and VCaP cells [79]. Thus, while there is controversy regarding NOX1 expression in prostate cancer cells, NOX1 expression seems to be absent or low in DU145 cells.

In animal experiments using nude mice, NOX1 overexpression increased tumorigenesis and tumor growth in DU145 human prostate cancer cells [80]. A similar result in an animal model has been reported by other investigators [81]. In the transgenic adenocarcinoma of the mouse prostate (TRAMP) mice, NOX1 expression was significantly higher in high-grade prostatic intraepithelial neoplasia (PIN) and cancer cells, than in low-grade PIN and normal prostate epithelial cells [82]. However, NOX1 acted as a potent trigger of angiogenesis via the upregulation of VEGF in a DU145 xenograft model [80]. Thus, animal experiments have shown that NOX1 is positively associated with tumorigenesis and malignant behavior in prostate cancer. Furthermore, in recent years, in vitro models using adenovirus vectors have shown that NOX1 plays a role in the cell death of prostate cancer cells [83], and NOX1 has been associated with metastatic potential in a series of cell lines developed from LNCaP [81].

In humans, NOX1 mRNA and protein are overexpressed in prostate cancer as compared to non-tumor prostate tissues [81,84]. However, according to the Human Protein Atlas repository version 12 (www.proteinatlas.org), high protein expression has been detected in both tumor cells and non-tumoral epithelium, and several studies in humans have reported that the NOX1 mRNA level did not significantly differ between benign and malignant prostate tissues [68,79]. Unfortunately, there are few reports on the relationship between NOX1 expression and pathological features in patients with prostate cancer. In the report by Arnold et al. [84], the high protein expression of NOX1 was not associated with any clinicopathological features, biochemical failure, or survival.

In contrast to NOX1, NOX2 mRNA expression has been detected in DU145, PC-3, and LNCaP cells, but not in normal cell lines [78]. mRNA levels of NOX2 were reported to be higher in PC-3, DU145, and VCaP cells than in the benign prostate cell lines, EP156T and RWPE1 [79]. However, other investigators have shown that mRNA expression of NOX2 was low or undetectable in three of these cancer cell lines (LNCaP, DU145, and PC-3) [68]. A similar observation has been reported in 17 patients with moderately to poorly differentiated prostate adenocarcinoma tissues [68]. According to the Human Protein Atlas repository version 12 (www.proteinatlas.org), NOX2 shows low protein expression in both tumor cells and non-tumoral epithelium, while it is prominently expressed in stromal tissues. As for mRNA expression, the NOX2 status in malignant prostate tissues is similar to that in benign tissues [79]. Thus, although several studies have shown that NOX2 expression is increased in prostate cancer as compared to non-tumoral tissues, opposite results have been shown by in vivo studies. Unfortunately, there is little information on the pathological significance of NOX2 expression in prostate cancer.

As mentioned above, the pathological significance of NOX3 in malignancies is not yet fully understood. NOX3 mRNA and protein expression was not observed in the prostate cancer cell lines, DU145, LNCaP, and PC-3 [68,77,78]. Protein expressions of NOX 1, 2, 4, and 5 have been detected in PC-3 cells, while NOX3 expression was absent [85]. Furthermore, no significant difference in NOX3 mRNA expression was detected between human prostate cancer tissues and non-tumoral tissues [68,79]. Although the information on NOX3 expression in prostate cancer is scarce, studies have shown that NOX3 has limited or no pathological significance in prostate cancer.

NOX4 is reportedly expressed in DU145, PC-3, and LNCaP cells, but not in a normal prostate cell line [78]. In addition, NOX4 mRNA levels in prostate cancer are significantly higher than those in benign prostate tissues [79]. In contrast, in a study by Juhasz et al., overexpression of NOX4 mRNA was not detected in the above three cell lines and human prostate cancer tissues [68]. According to the Human Protein Atlas repository version 12 (Available online: www.proteinatlas.org), tumor cells and non-tumoral epithelium show low NOX4 protein expression. Regarding its pathological roles, NOX4 is reported to contribute to angiogenesis via the upregulation of VEGF [65]. Interestingly, in rat prostate cancer, castration results in dramatic increases in NOX1, NOX2, and NOX4 [86]. Regarding the relationship between hormonal conditions and NOXs, NOX2 and NOX4 mRNA levels were upregulated by androgens and downregulated in the absence of androgens in the human androgen-responsive but not dependent prostate cell line, 22Rv1 [87]. This NOX-related mechanism, which involves ROS production, is associated with radiosensitivity in prostate cancer cells [87]. On the other hand, no significant change in NOX5 mRNA levels was observed upon castration in rats with prostate cancer [86]. In addition to androgens, adiponectin has induced a strong increase in NOX2 and NOX4 mRNA expression in DU145 and 22Rv1 human prostate cancer cells, which were NOX2-dominated and NOX4-dominated, respectively [88].

Kumar et al. reported NOX5 expression in prostate cancer cell lines (DU145, PC-3, and LNCaP cells), while it was not detected in a normal prostate cell line [78]. Other investigators detected NOX5 mRNA in benign prostate cancer cells (RWPE1) and cancer cell lines (LNCaP, VCaP, DU145, and PC-3), with the highest levels being observed in LNCaP and PC-3 cells [77,79]. From these results, it can be said that NOX5 mRNA is widely expressed in prostate cancer cell lines. Actually, NOX5 is held to be the most consistently expressed member of the NOX family in prostate cancer cell lines [79]. However, Juhasz et al. observed overexpression of NOX5 mRNA in PC-3, but not in DU145 cells [68]. Thus, there is a possibility that NOX5 mRNA expression may rely on androgens. Furthermore, a comparative analysis of NOX5 gene expression in tumor samples vs. adjacent non-malignant tissue showed no significant differences [68]. Brar et al. reported that, in human prostate tissues, NOX5 mRNA is widely expressed in both cancer and normal glands, based on which, the authors concluded that NOX5 mRNA expression is not a marker of malignant transformation [77]. Similar results have been reported for NOX5 protein expression in human tissues; NOX5 protein was expressed in 50 out of 62 human prostate cancer tissues (80.6%) in a study by Antony et al. [89]. Based on data in the Human Protein Atlas repository version 12 (Available online: www.proteinatlas.org), both tumor cells and non-tumoral epithelium highly express NOX5 protein. In addition, it has been reported that protein expression levels of NOX-5 are not significantly different between cancer and benign tissues [79]. On the other hand, decreased mRNA expression of NOX5 in prostate cancer, as compared to benign tissues, has been reported [79]. This finding suggested that NOX5 acts as a tumor suppressor in the carcinogenesis of prostate cancer. However, the relationship between NOX5 expression and carcinogenesis is not entirely clear, and more detailed and wider studies are necessary. Regarding the pathological roles of NOX5, several reports have shown that downregulation of NOX5 expression inhibits cell proliferation and tumor growth and induces apoptosis in PC cells [77,78,79,90]. In addition, in PC-3 cells, NOX5 silencing led to a significant increase in apoptosis via the stimulation of caspase-3 and -7 [79]. From these facts, NOX5 is thought to stimulate the cell survival of prostate cancer cells.

### 3.2. DUOX1 and 2 in Prostate Cancer

Overexpression of DUOX1 mRNA was not detected in the three prostate cancer cell lines, LNCaP, DU145, and PC-3 [63]. Other investigators reported that DUOX1 mRNA was detected at high levels in DU145 cells; however, such high expression was also detected in the benign prostate cell lines, EP156T and RWPE1 [79]. PC-3 cells express DUOX1 protein, but at a level similar to that in HeLa cells [85]. In humans, DUOX1 is highly expressed in both normal and prostate tumor tissues, and while some patients showed higher DUOX1 expression in tumoral tissues than in normal ones, the authors did not judge that the difference was significant [68]. According to the Human Protein Atlas repository version 12 (Available online: www.proteinatlas.org), DUOX1 protein expression is not detected in tumor cells and non-tumoral epithelium. On the other hand, it has been reported that DUOX1 mRNA expression in prostate cancer tissues is significantly lower than that in non-tumor tissues [79]. Thus, although DUOX1 expression has been detected in prostate cancer cells, its pathological significance is not fully understood.

DUOX2 mRNA expression has been reported to be low or undetectable in prostate cancer cell lines [68]. Other investigators detected high expression of DUOX2 mRNA in DU145 cells, but not in PC-3 cells [79]. Although DUOX2 expression has been observed in human cancer tissues, it is also highly expressed in normal tissues, indicating that DUOX2 is not overexpressed in human cancer tissues [68]. In addition, DUOX2 mRNA expression in prostate cancer tissues did not differ from that in non-tumoral tissues [68].

Several reports have shown pathological roles of DUOXs in prostate cancer. For example, BxPC-3 cells responded to interferon-γ treatment by upregulating DUOX2 protein [75]. There is a report that ROS levels in prostate cancer (PC-3 cells) are constitutively maintained by DUOX1 and 2, and these ROS lead to increased apoptosis resistance via positive regulation of Akt signaling [85].

### 3.3. NOXs and DUOXs in RCC

In contrast to prostate cancer, there is very limited information on the expression of NOXs in RCC. In an in vitro study, the activation of NOX1 and NOX4 maintained HIF-2α protein expression and thereby contributed to the tumorigenesis of RCC [91]. NOX4 expression in RCC cell lines was higher than that in a normal renal tubular cell line [91]. In vivo, inhibition of NOX4 expression by siRNA, abrogated tumorigenesis, cell invasion, and tumor growth in a murine xenograft model of RCC [92]. In a study by Chang et al., NOX4 contributed to the chemoresistance of RCC, by regulating apoptotic signaling, including anti-apoptotic B-cell lymphoma (Bcl)-XL and Bcl-2 and pro-apoptotic Bax [93]. Unfortunately, there are only a few reports on the expression and pathological roles of NOX2, 3, and 5 in RCC.

DUOX1 and 2 were reported to be highly expressed in both normal and RCC tissues [68]. However, there is no report with detailed information on the pathological significance of DUOXs in RCC. There is a general agreement that ROS-mediated mechanisms of oxidative stress play important roles in carcinogenesis, tumor development, and progression in RCC [94]. Specifically, angiogenesis is one of the important processes for tumor growth, cell dissemination, and prognosis in RCC. Unfortunately, there is no report on the angiogenic roles of NOXs. Therefore, more detailed and wider studies are necessary to discuss the pathological characteristics of this disease.

### 3.4. NOXs and DUOXs in Urothelial Cancer

Similar to RCC, the information on NOXs in urothelial cancer is insufficient to discuss their pathological roles in tumorigenesis and malignant aggressiveness. NOX1 protein expression is higher in high-grade and invasive disease than in low-grade and non-invasive disease, in human bladder cancer tissues [49]. Unfortunately, additional results on NOX1 expression in urothelial cancer have not yet been reported. However, several reports suggest that NOX1 is associated with tumor development and apoptosis in urothelial cancer. For example, the silencing of ALKBH8, a member of the human AlkB family of DNA repair molecules [95], leads to a decrease in ROS production, via the downregulation of NOX1 in urothelial cancer and to apoptosis resistance, resulting in bladder cancer development [49]. In addition, other reports have shown that the knockdown of NOX1 reduces ROS production and apoptosis by FK228, a histone deacetylase inhibitor, in a human bladder cancer cell line (J82 cells) [96].

Overexpression of NOX2 has been detected in urothelial cancer cells, whereas expression in normal urothelial cells is low, suggesting that NOX2 may play a crucial role in the carcinogenesis of urothelial cancer [97]. However, this study also showed that NOX2 expression was not significantly associated with grade or pT stage in 93 patients with urothelial cancer [97]. In another report, the DNA repair molecule, ALKBH3, was positively associated with urothelial cancer cell survival, through NOX2-dependent ROS production [97]. Thus, NOX2 is speculated to be required for tumor growth in urothelial cancer.

NOX4 is highly expressed in the urothelial cancer cell lines, T24, UMUC6, and KK47 [93]. In addition, NOX4 overexpression was detected in the cancer tissues of patients with low- or high-grade and non-invasive or invasive urothelial cancers, including carcinoma in situ, as compared to normal urothelium [98]. However, its level was not significantly correlated with the grade, pT stage, or tumor growth in 82 patients with urothelial cancer [98]. Although pathological roles of NOX4 expression were not speculated to be important, the authors observed significantly higher NOX4 immunostaining in the percutaneous lesion dysplasia than in normal urothelium [98]. Regarding apoptosis, NOX4 gene silencing did induce a change in the apoptotic activity of urothelial cancer cells [49]. Conversely, leukotriene B4 receptor 2 regulates cell invasion and metastasis by ROS production in urothelial cancer, and NOX1 and NO4 are associated with the mechanism of this phenomenon [99].

To our knowledge, there are no reports on the expression of NOX3 and NOX5 in urothelial cancer. We emphasize the importance for further investigation into the expression and pathological roles of NOX3 and NOX5 in urothelial cancer. Furthermore, we should note that the discussion of the biological activities and pathological significances of NOX family members was based on mRNA expression in cell culture. In other words, there is less information on the protein expression of the NOX family in human cancer tissues because of the lack of reliable antibodies. In recent years, specific antibodies for NOX family members have been developed. Wider and more detailed studies are expected to clarify the pathological significance and prognostic roles of these enzymes in urological cancers. We summarized pathological roles of NOX family in prostate cancer, renal cell cancer, and urothelial cancer in Table 1.

## 4. Angiogenesis and ROS

Proliferation, migration, and tube formation of endothelial cells are well known to be important steps of cancer-related angiogenesis. ROS can modulate tumor angiogenesis via the regulation of these steps [28]. VEGF-A is well known as one of the strongest pro-angiogenic factors under physiological and pathological conditions. Numerous studies have reported a positive correlation between VEGF-A expression and angiogenesis in cancer tissues evaluated by micro-vessel density [100,101]. Although various pathways have been reported to be regulators of VEGF-A expression, ROS are also known to be a representative VEGF-A-related factor under pathological conditions, including malignancies [102,103,104]. In addition to VEGF-A, information on hypoxia-induced changes is essential for a discussion of the pathological roles of tumor angiogenesis in urological cancers, especially in RCC. Hypoxia can also affect ROS production and this is recognized as a regulator of angiogenesis in these cancers [105,106,107]. Therefore, several investigators have paid special attention to the relationship between angiogenesis and ROS production in malignant tumors. In fact, detailed molecular regulatory mechanisms of angiogenesis, induced by ROS under pathological conditions, including malignancies, have been described in previous excellent reviews [28,108,109]. In this review, we demonstrate the factors modulating angiogenesis by ROS production in urological cancers.

### 4.1. Angiogenesis and ROS in Prostate Cancer

Various types of chemical agents, chemokines, and natural compounds can mediate carcinogenesis, malignant behavior, and treatment response, via the regulation of tumor angiogenesis. In addition, ROS are often associated with angiogenesis-related mechanisms in prostate cancer. In this section, we describe representative examples of factors that produce ROS and thus affect angiogenesis in prostate cancer.

Arsenite, an environmental toxicant, widely distributed in water, food, and air, acts as a carcinogen for prostate cancer, via the activation of PI3K/Akt signaling and the expression of VEGF and HIF-1α. ROS have been associated with this mechanism in DU145 cells [110]. In addition, Ampelopsin (dihydromyricetin), a natural flavonoid, is suggested to be a useful agent for the prevention and treatment of prostate cancer, and suppression of ROS and angiogenesis is thought to underlie its anti-cancer effects [111]. Similar effects of other flavonoids have been reported in the androgen-independent prostate cancer cell line, PC-3 [112].

Regarding chemical agents, vanadate, known as sodium orthovanadate, is associated with carcinogenesis for prostate cancer, via angiogenesis- and ROS-related mechanisms [105]. Conversely, when the anti-cancer effects of a combination therapy with paclitaxel and S13 (a tyrosine kinase inhibitor with a prevalent specificity for Src) were analyzed in a hormone-insensible prostate cancer cell model, tumor growth and angiogenesis were suppressed by the concomitant impairment of endothelial migration and VEGF production [113]. Interestingly, significant effects were observed in combination therapy versus control and single treatments, and a reduction in ROS production was similarly found only in combination therapy [113]. In brief, the combination of paclitaxel and S13 may strongly inhibit tumor angiogenesis, via the reduction of ROS production. In addition to anti-cancer agents, ROS are associated with interleukin-6-related radio-sensitivity and tumor angiogenesis in animal models [114]. However, angiogenesis is suppressed by exosomes derived from menstrual stem cells through a reduction in the secretion/activity of pro-angiogenic molecules, such as VEGF and NF-κB, and ROS inhibition is associated with this mechanism in prostate cancer [115]. Thus, ROS plays important roles in angiogenesis, caused by various stimuli, in prostate cancer.

### 4.2. Angiogenesis and ROS in RCC and Urothelial Cancer

As mentioned above, as angiogenesis is the most important step in tumor growth and progression in RCC, anti-angiogenetic agents are useful for angiogenesis treatment. In fact, hypoxia induces ROS production, and subsequently activates RhoA, which leads to angiogenesis through HIF-1α induction and VEGF production in RCC [106]. Unfortunately, the information about ROS-induced angiogenesis in RCC is very limited. In regard to natural compounds, piperlongumine—an alkaloid present in the fruit of the long pepper (*Piper longum*)—and its analogs, rapidly reduced protein and mRNA levels of c-Met, a regulator of angiogenesis, via a ROS-dependent mechanism in the RCC cell lines, 786-0 and PNX0010 [116]. However, the combination of quercetin (a flavonoid) and hyperoside (an organic compound) was reported to decrease ROS production by up to 2.25-fold, which correlated with angiogenesis in 786-0 cells [117].

Similar to RCC, there are few reports on ROS-induced angiogenesis in urothelial cancer. Platinum agents, such as cisplatin and carboplatin, are key drugs for the treatment of patients with advanced urothelial cancer. Cis-dichlorodiammineplatinum has been reported to increase ROS production in the urothelial cancer cell lines, T24, KU-1, and KU-19-19. In addition, it upregulates angiotensin II type 1 receptor (AT1R) expression though ROS production and enhances VEGF production [118]. Conversely, AT1R expression has been reported to be significantly associated with microvessel density in non-muscle-invasive bladder cancer [119]. The same research group showed that AT1R signaling was upregulated when tumors progressed after cisplatin-based regimens, and there was increased ROS production related to this phenomenon in bladder cancer [118]. The knockdown of ALKBH3 led to the downregulation of angiogenesis, ROS production, and VEGF expression in bladder cancer, in vitro and in vivo, in an orthotopic mouse model [97]. In addition to ALKBH3, ALKB8 is reported to be significantly associated with tumor grade, cancer cell invasion, and prognosis, via the regulation of angiogenesis and ROS production in urothelial cancer [49]. Other investigators have reported that MMP-1, which is associated with cell invasion and metastasis, is upregulated under hypoxic conditions. Such hypoxia-induced changes in MMP-1 expression are accompanied by the stabilization of HIF-1α and -2α and a rise in intracellular ROS in metastatic 253J-BV cells [107]. The authors concluded that ROS play important roles in hypoxia-mediated MMP-1 expression, and that HIF stabilization under hypoxia is dependent on increases in intracellular ROS levels.

## 5. Apoptosis and ROS

Apoptosis or programmed cell death is executed via two major pathways: the extrinsic (death receptor-dependent) and the intrinsic (mitochondrial) pathway. In the extrinsic pathway, apoptosis is regulated by the binding of death-inducing ligand. For example, the interaction between Fas ligand (FasL) and its receptor, Fas receptor (FasR), leads to the activation of the caspase cascade and the induction of apoptosis. Apoptosis via the caspase cascade can be also stimulated by the ligation of tumor necrosis factor (TNF)-α and TNF-related apoptosis-inducing ligand (TRAIL) to their respective receptors, such as TNF receptor and death receptors. The intrinsic pathway is not necessarily initiated by intracellular effects, but may be initiated by outside triggers as well [120,121]. However, apoptosis by the intrinsic pathway is often initiated by increased mitochondrial membrane permeability. In short, pro-apoptotic factors, such as cytochrome-c and apoptosis-inducing factor, are released from the mitochondria though the mitochondrial permeability transition pore. The intrinsic pathway of apoptosis is tightly regulated by the balance of the B-cell lymphoma 2 (Bcl-2) family, composed of pro- and anti-apoptotic Bcl-2 proteins. As pro-apoptotic members, Bax, Bad, and Bid, are well known. Bcl-2, Bcl-cl, and Mcl-1 are representative anti-apoptotic factors. These members of the Bcl-2 family are regulated by ROS, via direct and indirect mechanisms [122,123]. Detailed apoptotic mechanisms induced by ROS under pathological conditions have been described in previous reviews [124,125]. Therefore, we here pay special attention to natural products and chemical agents that produce ROS and consequently modulate apoptosis in urothelial cancers.

### 5.1. ROS and Apoptosis in Prostate Cancer

Total flavonoids extracted from persimmon leaves, which are used as a herbal medicine in Asia, have been recently reported to induce mitochondrial apoptosis, via the inactivation of Bcl-2, upregulation of Bax, and release of cytochrome c in the androgen-independent prostate cancer cell line, PC-3 [126,125]. In addition to these mitochondria-related pathways, flavonoids induced apoptosis by ROS production [126]. Anethole, which is a major constituent of *Foeniculum vulgare* (fennel) essential oil and is widely used in folk medicine, inhibited cell proliferation, migration, and colony formation and induced apoptosis and cell arrest by ROS production [127]. Actually, numerous studies have shown that the induction of apoptosis by natural products reduced ROS production in prostate cancer cells. Because of space constraints, we introduce only the most recent reports published in 2017; *Punica granatum* (pomegranate) peel in mouse prostate cancer cells (TRAMP-C1) [128]; a new nanoemulsion system of rutin in PC-3 [129]; benzyl isothiocyanate in cruciferous plants in CRW-22Rv1 and PC-3 [130]; curcumin and resveratrol in LNCaP and PC-3 cells [131]; Chikusetsu saponin Iva, isolated from *Aralia taibaiensis* in PC-3 [132]; chrysin, a natural flavone found in numerous plant extracts, honey, and propolis, in DU145 and PC-3 cells [133]; lasalocid, an antibiotic from the group of carboxylic ionophores produced by *Streptomyces lasaliensis*, in PC-3 [134]; naringenin, an anti-oxidant flavonoid derived from citrus, in PC-3 [135]; and coumestrol, a major phytoestrogen abundant in soybeans, legumes, Brussels sprouts, and spinach, in LNCaP and PC-3 cells [136]. Furthermore, treatment with WZ35, a chemical analog of curcumin, induced apoptosis in two prostate cancer cell lines (RM-1 and DU145), and this anti-cancer effect depended on ROS production [137].

In addition to natural products, various chemicals have been reported to modulate apoptosis via ROS production. For example, salinomycin, a promising anti-cancer drug, induced apoptosis via ROS production, and this mechanism was related to ROS-mediated autophagy through the regulation of the PI3K/Akt/ mechanistic target of rapamycin (mTOR) and ERK/p38 MAPK signaling pathways [138]. In addition, toyocamycin, an antibiotic agent isolated from *Streptomyces* species, enhanced the apoptosis of PC-3 cells by the ROS-mediated signaling pathway, ERK/p38 MAPKs [139]. Conversely, there are several reports on the relationship between ROS production and treatment efficacy in prostate cancer. For example, treatment with JS-K, a glutathione S transferase-activated nitric oxide donor prodrug, for 24 h, increased the proportion of apoptotic cells, by inducing ROS production in prostate cancer cells (22RV1, C4-2, LNCaP, and PC-3) [140]. Interestingly, these authors also showed that the pro-apoptotic effect of JS-K is dose-dependent, and 22RV1 and C4-2 cells were more sensitive than LNCaP and PC-3 cells [141]. Furthermore, although selenite had a partial pro-apoptotic effect and carmustine showed no apoptosis induction in EGF-stimulated PC-3 cells, combination treatment with carmustine and selenite dramatically induced apoptosis in EGF-stimulated PC-3 cells [141]. This combination treatment increased ROS production, which triggered apoptosis in 22RV1 and PC-3 cells [141,142]. Based on these facts, this combination treatment was speculated to induce apoptosis via ROS production in prostate cancer cells. Similar findings have reported for the combination of orlistat, an anti-obesity drug, and 5-aminoimidazole-4-carboxamide ribonucleotide, an analog of adenosine monophosphate (AMP) that is capable of stimulating AMP-dependent protein kinase (AMPK) activity [143].

### 5.2. Apoptosis and ROS in RCC

Similar to prostate cancer, various substances and agents are associated with apoptosis via ROS production in RCC. Various chemical compounds have been shown to induce ROS production and to modulate apoptosis in RCC cells. Galangin, a flavonoid extracted from the root of *Alpinia officinarum*, induced apoptosis by increasing the intracellular concentration of ROS [144]. Eupatilin, a pharmacologically active component found in *Artemisia asiatica,* induced apoptosis in 786-O cells by ROS-mediated activation of the MAPK signaling pathway and inhibition of the PI3K/Akt signaling pathway [145]. Additionally, carnosic acid, the major bioactive compound of *Rosmarinus officinalis* L., is reported to induce apoptosis via a ROS-related mechanism in RCC cells (Caki cells) [146]. Thus, several natural compounds have been reported to be associated with ROS-induced apoptosis in RCC cells. We suggest that further studies are necessary because the number of studies on RCC are lower than those on prostate cancer.

A variety of chemical agents have been reported to induce apoptosis via ROS-related mechanisms. For example, the anti-hepatitis drug, bicyclol (4,4′-dimethoxy-5,6,5′,6′-bis(methylenedioxy)-2-hydroxymethy-l-2′-methoxy-carbonyl biphenyl, reportedly induces apoptosis and cell-cycle arrest, which depended on ROS production in RCC cells [147]. Furthermore, niclosamide, an anthelmintic drug, especially used for the treatment of tapeworm infection, can inhibit cell proliferation and induce apoptosis in RCC cell lines, and Wnt/β-catenin activities are associated with these anti-cancer effects [148]. The study also showed that niclosamide induces mitochondrial dysfunction, resulting in increased ROS levels [148]. These results support the possibility that existing therapeutic drugs for other diseases may be useful as new treatment strategies in patients with RCC. In fact, the combination of chloroquine and ABT-737, a small-molecule BH3 mimetic with very high affinity to Bcl-2, Bcl-xL, and Bcl-w, which induces apoptosis by inhibiting pro-survival Bcl-2 proteins and activating caspases, synergistically decreased RCC cell viability as compared to treatment with a single reagent, and the level of ROS was increased after treatment with ABT-737 and chloroquine [149]. On the other hand, sorafenib, a multikinase inhibitor approved for the treatment of advanced RCC, induces apoptosis by ROS production, and such ROS-dependent apoptotic processes are independent of caspase activities and Bcl-2 family proteins, including bax and bak; however, sorafenib-induced ROS accumulation mediates increased caspase-8 activation [150]. These findings may help to improve the anti-cancer effects of sorafenib-based therapy.

ROS negatively regulates cellular FLICE-inhibitory protein (c-FLIP) via proteasomal degradation, leading to the induction of apoptosis via the extrinsic pathway [151]. In RCC cells, the anti-cancer alkaloid, berberine, sensitized TRAIL-induced apoptosis, through the downregulation of c-FLIP in renal cancer cells [152]. Similarly, 6-shogaol, a potent bioactive compound in ginger, enhanced TRAIL-mediated apoptosis in Caki RCC cells, via ROS-mediated cytochrome c release and downregulation of c-FLIP expression [153]. Similar effects in Caki cells have been reported for thymoquinones—phytochemical compounds found in the plant Nigella sativa [154]. Thus, ROS can modulate apoptosis, through direct and indirect mechanisms, in RCC cells.

### 5.3. Apoptosis and ROS in Urothelial Cancer

Compared to prostate cancer and RCC, less information is available on regulators of ROS-induced apoptosis in urothelial cancer. Guizhi Fuling Wan, a traditional Chinese medicine, suppressed cell proliferation and induced apoptosis in bladder cancer cells, and the authors speculated that one possible mechanism underlying these effects is an increase in intracellular ROS, leading to the activation of the ATM/CHK2 and ATM/P53 pathways [155]. Furthermore, a high concentration of aristolochic acid, extracted from species of *Aristolochia*, induced cell death, partly via apoptosis, activated via increased ROS production [156]. In addition, dioscin, a natural steroid saponin, and thymol, a phenolic compound, triggered ROS-induced apoptosis in T24 bladder cancer cells [157].

Regarding chemical agents, *O*2-(2,4-dinitrophenyl) 1-[(4-ethoxycarbonyl)piperazin-1-yl]diazen-1-ium-1,2-diolate (JS-K) suppressed cell proliferation and induced the apoptosis of bladder cancer cells, in a concentration-dependent manner, by increasing ROS levels [158]. Furthermore, 5-bromo-3-(3-hydroxyprop-1-ynyl)-2*H*-pyran-2-one has been reported to induce apoptosis, via the activation of caspases and increased ROS production [159]. Conversely, chloroquine reportedly induced ROS production and affected cell death in prostate cancer cells, but not in bladder cancer cells [160,161]. Thus, the activity for ROS-induced apoptosis depends on the type of cancer.

## 6. Conclusions

This review mainly discussed the relationships between ROS and malignant potential in urological cancers. In particular, we paid special attention to the NOX family, angiogenesis, and apoptosis, which are key factors for carcinogenesis, tumor growth, and the cell dissemination of prostate cancer, renal cell carcinoma, and urothelial cancer. There is no general agreement on the overexpression of NOX family members in prostate cancer. On the other hand, the information on expression of NOXs in RCC and urothelial cancer is insufficient to draw definitive conclusions. However, NOX3 does not seem to play an important role in malignant aggressiveness in all urological cancers. Thus, readers may notice that the information on the pathological significance of oxidative stress is insufficient to discuss observations and treatment strategies based on ROS in urological cancers, especially in RCC and urothelial cancer. We emphasize that further investigations are necessary to understand the biological and pathological characteristics of urological cancers as vascular diseases. Once such detailed information is available, there is a possibility that the NOX family may provide useful predictive factors and/or potential therapeutic targets for patients with these types of cancer.

## Figures and Tables

**Table 1:** Summary of pathological roles of NOXs in urological cancers.

Characteristics as Malignant Cells	Types	NOX					DUOX		References
		1	2	3	4	5	1	2	
Overexpression in cancer cells	PCa	Y/N	Y/N	N	Y/N	Y/N	N	N	[68,77,78,79,80,81,82,84,85,89]
	RCC	N	N	N	Y/N	N	N	N	[68,91]
	UC	–	Y	–	Y	–	–	–	[97,98]
Tumorigenesis	PCa	Y	–	–	–	–	–	–	[80,81]
	RCC	Y	–	–	Y	–	–	–	[91,92]
	UC	–	–	–	–	–	–	–	–
Cell death/Apoptosis	PCa	Y	–	–	–	Y	Y	Y	[77,79,83,85]
	RCC	–	–	–	Y	–	–	–	[93]
	UC	Y	–	–	N	–	–	–	[49,96]
Angiogenesis/Angiogenesis-related factors	PCa	Y	–	–	Y	–	–	–	[65,80]
	RCC	–	–	–	–	–	–	–	–
	UC	–	N	–	–	–	–	–	[97]
Gleason score/Grade	PCa	N	–	–	–	–	–	–	[84]
	RCC	–	–	–	–	–	–	–	–
	UC	Y	N	–	N	–	–	–	[49,97,98]
T stage/Tumor growth/Invasion	PCa	Y/N	Y	–	Y	Y	–	–	[65,77,78,79,80,82,84]
	RCC	–	–	–	Y	–	–	–	[92]
	UC	Y	Y/N	–	Y/N	–	–	–	[49,97,98,99]
N stage/M stage/Metastasis	PCa	Y/N	–	–	–	–	–	–	[81,84]
	RCC	–	–	–	–	–	–	–	–
	UC	Y	–	–	Y	–	–	–	[99]
Outcome/Survival	PCa	N	–	–	–	–	–	–	[84]
	RCC	–	–	–	–	–	–	–	–
	UC	–	–	–	–	–	–	–	

NOX; NADPH oxidases, DUOX; dual oxidases, Y; Yes, N; No, PCa; prostate cancer, RCC; renal cell carcinoma, UC; urothelial cancer.

## References

[1] Sies H. (1997). Oxidative stress: Oxidants and antioxidants. Exp. Physiol..

[2] Goodman M., Bostick R.M., Dash C., Terry P., Flanders W.D., Mandel J. (2008). A summary measure of pro- and anti-oxidant exposures and risk of incident, sporadic, colorectal adenomas. Cancer Causes Control.

[3] Pizzino G., Irrera N., Cucinotta M., Pallio G., Mannino F., Arcoraci V., Squadrito F., Altavilla D., Bitto A. (2017). Oxidative Stress: Harms and Benefits for Human Health. Oxid. Med. Cell. Longev..

[4] Andrisic L., Dudzik D., Barbas C., Milkovic L., Grune T., Zarkovic N. (2017). Short overview on metabolomics approach to study pathophysiology of oxidative stress in cancer. Redox Biol..

[5] Carini F., Mazzola M., Rappa F., Jurjus A., Geagea A.G., Al Kattar S., Bou-Assi T., Jurjus R., Damiani P., Leone A. (2017). Colorectal carcinogenesis: Role of oxidative stress and antioxidants. Anticancer Res..

[6] Schieber M., Chandel N.S. (2014). ROS function in redox signaling and oxidative stress. Curr. Biol..

[7] D’Autréaux B., Toledano M.B. (2007). ROS as signaling molecules: Mechanisms that generate specificity in ROS homeostasis. Nat. Rev. Mol. Cell Biol..

[8] Valko M., Leibfritz D., Moncol J., Cronin M.T., Mazur M., Telser J. (2007). Free radicals and antioxidants in normal physiological functions and human disease. Int. J. Biochem. Cell Biol..

[9] Halliwell B. (2013). The antioxidant paradox: Less paradoxical now?. Br. J. Clin. Pharmacol..

[10] Brown D.I., Griendling K.K. (2015). Regulation of signal transduction by reactive oxygen species in the cardiovascular system. Circ. Res..

[11] Trachootham D., Alexandre J., Huang P. (2009). Targeting cancer cells by ROS-mediated mechanisms: A radical therapeutic approach?. Nat. Rev. Drug Discov..

[12] Du M.Q., Carmichael P.I., Phillips D.H. (1994). Induction of activating mutations in the human c-Ha-ras-a proto-oncogene by oxygen free radicals. Mol. Carcinog..

[13] Ushijima T. (2005). detection and interpretation of altered methylation patterns in cancer cells. Nat. Rev. Cancer..

[14] Toyokuni S. (2008). Molecular mechanisms of oxidative stress-induced carcinogenesis: From epidemiology to oxygenomics. IUBMB Life.

[15] Lee K.H., Kim S.W., Kim J.R. (2009). Reactive oxygen species regulate urokinase plasminogen activator expression and cell invasion via mitogen-activated protein kinase pathways after treatment with hepatocyte growth factor in stomach cancer cells. J. Exp. Clin. Cancer Res..

[16] Liou G.Y., Storz P. (2010). Reactive oxygen species in cancer. Free Radic. Res..

[17] Li Q., Engelhardt J.F. (2006). Interleukin-1β induction of NFκB is partially regulated by H_2_O_2_-mediated activation of NFκB-inducing kinase. J. Biol. Chem..

[18] Fresno Vara J.A., Casado E., de Castro J., Cejas P., Belda-Iniesta C., González-Barón M. (2004). PI3K/Akt signaling pathway and cancer. Cancer Treat. Rev..

[19] Kwon J., Lee S.R., Yang K.S., Ahn Y., Kim Y.J., Stadtman E.R., Rhee S.G. (2004). Reversible oxidation and inactivation of the tumor suppressor PTEN in cells stimulated with peptide growth factor. Proc. Natl. Acad. Sci. USA.

[20] Saybasilli H., Yülsel M., Haklar G., Yalçin A.S. (2001). Effect of mitochondrial electron transport chain inhibitors on superoxide radical generation in rat hippocampal and striatal slices. Antioxid. Redox Signal..

[21] Li X., Fang P., Mai J., Choi E.T., Wang H., Yang X.F. (2013). Targeting mitochondrial reactive oxygen species as novel therapy for inflammatory diseases and cancers. J. Hematol. Oncol..

[22] Segal A.W., Shatwell K.P. (1997). The NAPDH oxidase of phagocytic leukemia. Ann. N. Y. Acad. Sci..

[23] Kröller-Schön S., Steven S., Kossmann S., Scholz A., Daub S., Oelze M., Xia N., Hausding M., Mikhed Y., Zinssius E. (2015). Molecular mechanisms of the crosstalk between mitochondria and NADPH oxidase through reactive oxygen species-studies in white blood cells and in animal models. Antioxid. Redox Signal..

[24] Korbecki J., Baranowska-Bosiacka I., Gutowska I., Chlubek D. (2013). The effect of reactive oxygen species on the synthesis of prostanoids from arachidonic acid. J. Physiol. Pharmacol..

[25] Nelson K.K., Melendez J.A. (2004). Mitochondrial redox control of matrix metalloproteinases. Free Radic. Biol. Med..

[26] Siwik D.A., Colucci W.S. (2004). Regulation of matrix metalloproteinases by cytokines and reactive oxygen/nitrogen species in the myocardium. Heart Fail. Rev..

[27] Fiaschi T., Cozzi G., Raugei G., Formigli L., Ramponi G., Chiarugi P. (2006). Redox regulation of β-actin during integrin-mediated cell adhesion. J. Biol. Chem..

[28] Galadari S., Rahman A., Pallichankandy S., Thayyullathil F. (2017). Reactive oxygen species and cancer paradox: To promote or to suppress?. Free Radic. Biol. Med..

[29] Leufkens A.M., van Duijnhoven F.J., Woudt S.H., Siersema P.D., Jenab M., Jansen E.H., Pischon T., Tjønneland A., Olsen A., Overvad K. (2012). Biomarkers of oxidative stress and risk of developing colorectal cancer: A cohort-nested case-control study in the European Prospective Investigation Into Cancer and Nutrition. Am. J. Epidemiol..

[30] Tochhawng L., Deng S., Pervaiz S., Yap C.T. (2013). Redox regulation of cancer cell migration and invasion. Mitochondrion.

[31] Prasad S., Gupta S.C., Tyagi A.K. (2017). Reactive oxygen species (ROS) and cancer: Role of antioxidative nutraceuticals. Cancer Lett..

[32] Toyokuni S., Okamoto K., Yodoi J., Hiai H. (1995). Persistent oxidative stress in cancer. FEBS Lett..

[33] Schumacker P.T. (2015). Reactive oxygen species in cancer: A dance with the devil. Cancer Cell..

[34] Morry J., Ngamcherdtrakul W., Yantasee W. (2017). Oxidative stress in cancer and fibrosis: Opportunity for therapeutic intervention with antioxidant compounds, enzymes, and nanoparticles. Redox Biol..

[35] Szatrowski T.P., Nathan C.F. (1991). Production of large amounts of hydrogen peroxide by human tumor cells. Cancer Res..

[36] Panieri E., Santoro M.M. (2016). ROS homeostasis and metabolism: A dangerous liason in cancer cells. Cell Death Dis..

[37] Lambeth J.D., Kawahara T., Diebold B. (2007). Regulation of Nox and Duox enzymatic activity and expression. Free Radic. Biol. Med..

[38] Jiang F., Zhang Y., Dusting G.J. (2011). NADPH oxidase-mediated redox signaling: Roles in cellular stress response, stress tolerance, and tissue repair. Pharmacol. Rev..

[39] Gào X., Schöttker B. (2017). Reduction-oxidation pathways involved in cancer development: A systematic review of literature reviews. Oncotarget.

[40] Block K., Gorin Y. (2012). Aiding and abetting roles of NOX oxidases in cellular transformation. Nat. Rev. Cancer.

[41] Suh Y.A., Arnold R.S., Lassegue B., Shi J., Xu X., Sorescu D., Chung A.B., Griendling K.K., Lambeth J.D. (1999). Cell transformation by the superoxide-generating oxidase MOX1. Nature.

[42] Arnold R.S., Shi J., Murad E., Whalen A.M., Sun C.Q., Polavarapu R., Parthasarathy S., Petros J.A., Lambeth J.D. (2001). Hydrogen peroxide mediates the cell growth and transformation caused by the mitogenic oxidase Nox1. Proc. Natl. Acad. Sci. USA.

[43] Böhm B., Heinzelmann S., Motz M., Bauer G. (2015). Extracellular localization of catalase is associated with the transformed state of malignant cells. Biol. Chem..

[44] Heinzelmann S., Bauer G. (2010). Multiple protective functions of catalase against intercellular apoptosis inducing ROS signaling of human tumor cells. Biol. Chem..

[45] Bauer G. (2015). Increasing the endogenous NO level causes catalase inactivation and reactivation of intercellular apoptosis signaling specifically in tumor cells. Redox Biol..

[46] Bánfi B., Muturana A., Jaconi S., Arnaudeau S., Laforge T., Sinha B., Ligeti E., Demaurex N., Krause K.H. (2000). A mammalian H^+^ channel generated trough alternative splicing of the NADPH oxidase homolog NOH-1. Science.

[47] Kobayashi S., Nojima Y., Shibuya M., Maru Y. (2004). Nox1 regulates apoptosis and potentially stimulates branching morphogenesis in sinusoidal endothelial cells. Exp. Cell Res..

[48] Shinihara M., Shang W.H., Kubodera M., Harada S., Mitsushita J., Kato M., Miyazaki H., Sumimoto H., Kamata T. (2007). Nox1 redox signaling mediates oncogenic Ras-induced disruption of stress fibers and focal adhesion by down-regulating Rho. J. Biol. Chem..

[49] Shimada K., Nakamura M., Anai S., De Velasco M., Tanaka M., Tsujikawa K., Ouji Y., Konishi N. (2009). A novel human AlkB homologue, ALKBH8, contributes to human bladder cancer progression. Cancer Res..

[50] De Carvalbo D.D., Sadok A., Bourgarel-Rey V., Gattacceca F., Penel C., Lehmann M., Kovacic H. (2008). Nox1 downstream of 12-lipoxygenase controls cell proliferation but not cell spreading of colon cancer. Int. J. Cancer.

[51] Raad H., Serrano-Sanchez M., Harfouche G., Mahfouf W., Bortolotto D., Bergeron V., Kasraian Z., Dousset L., Hosseini M., Taieb A. (2017). NADPH oxidase-1 plays a key role in keratinocyte responses to UV radiation and UVB-induced skin carcinogenesis. J. Investig. Dermatol..

[52] Bhardwaj V., Gokulan R.C., Horvat A., Yermalitskaya L., Korolkova O., Washington K.M., El-Rifai W., Dikalov S.I., Zaika A.I. (2017). Activation of NADPH oxidases leads to DNA damage in esophageal cells. Sci. Rep..

[53] Aydin E., Johansson J., Nazir F.H., Hellstrand K., Martner A. (2017). Role of NOX2-derived reactive oxygen species in NK cell-mediated control of murine melanoma metastasis. Cancer Immunol. Res..

[54] Aurelius J., Hallner A., Werlenius O., Riise R., Möllgård L., Brune M., Hansson M., Martner A., Thorén F.B., Hellstrand K. (2017). NOX2-dependent immunosuppression in chronic myelomonocytic leukemia. J. Leukoc. Biol..

[55] Montalvo-Javé E.E., Olguín-Martínez M., Hernández-Espinosa D.R., Sánchez-Sevilla L., Mendieta-Condado E., Contreras-Zentella M.L., Oñate-Ocaña L.F., Escalante-Tatersfield T., Echegaray-Donde A., Ruiz-Molina J.M. (2016). Role of NADPH oxidases in inducing a selective increase of oxidant stress and cyclin D1 and checkpoint 1 over-expression during progression to human gastric adenocarcinoma. Eur. J. Cancer..

[56] Bánfi B., Malgrange B., Knisz J., Steger K., Steger K., Dubois-Dauphin M., Krause K.H. (2004). NOX3, a superoxide-generating NADPH oxidase of the inner ear. J. Biol. Chem..

[57] Wang Y., Liu Q., Zhao W., Zhou X., Miao G., Sun C., Zhang H. (2017). NADPH oxidase activation contributes to heavy ion irradiation-induced cell death. Dose Response.

[58] Leung E.L., Fan X.X., Wong M.P., Jiang Z.H., Liu Z.Q., Yao X.J., Lu L.L., Zhou Y.L., Yau L.F., Tin V.P. (2016). Targeting tyrosine kinase inhibitor-resistant non-small cell lung cancer by inducing epidermal growth factor receptor degradation via methionine 790 oxidation. Antioxid. Redox Signal..

[59] Cheng G., Cao Z., Xu X., van Meir E.G., Lambeth J.D. (2001). Homologs of gp91phox: Cloning and tissue expression of Nox3, Nox4, and Nox5. Gene.

[60] Lee J.K., Edderkaoui M., Truong P., Ohno I., Jang K.T., Berti A., Pandol S.J., Gukovskaya A.S. (2007). NADPH oxidase promotes pancreatic cancer cell survival via inhibiting JAK2 dephosphorylation by tyrosine phosphatases. Gastroenterology.

[61] Maloney E., Sweet I.R., Hockenbery D.M., Pharm M., Rizzo N.O., Tateya S., Handa P., Schwartz M.W., Kim F. (2009). Activation of NF-κB by palmitate in endothelial cells, a key role for NADPH oxidase-derived superoxide in response to TLR4. Activation.

[62] Liu R.-M., Choi J., Wu J.-H., Gaston Pravia K.A., Lewis K.M., Brand J.D., Mochel N.S., Krzywanski D.M., Lambeth J.D., Hagood J.S. (2010). Oxidative modification of nuclear mitogen-activated protein kinase phosphatase 1 is involved in transforming growth factor β1-induced expression of plasminogen activator inhibitor 1 in fibroblasts. J. Biol. Chem..

[63] Brar S.S., Kennedy T.P., Whorton A.R., Sturrock A.B., Huecksteadt T.P., Ghio A.J., Hoidal J.R. (2001). Reactive oxygen species from NAD(P)H: Quinone oxidoreductase constitutively activate NF-κB in malignant melanoma cells. Am. J. Physiol..

[64] Vaquero E.C., Edderkaoui M., Pandol S., Gukovsky I., Gukovskaya A.S. (2004). Reactive oxygen species produced by NAD(P)H oxidase inhibit apoptosis in pancreatic cancer cells. J. Biol. Chem..

[65] Xia C., Meng Q., Liu L.Z., Rojanasakul Y., Wang X.R., Jiang B.H. (2007). Reactive oxygen species regulate angiogenesis and tumor growth through vascular endothelial growth factor. Cancer Res..

[66] Datla S.R., Peshavariya H., Dusting G.J., Mahadev K., Goldstein B.J., Jiang F. (2007). Important role of Nox4 type NADPH oxidase in angiogenic responses in human microvascular endothelial cells in vitro. Arterioscler. Thromb. Vasc. Biol..

[67] Vallet P., Charnay Y., Steger K., Ogier-Denis E., Kovari E., Herrmann F., Michel J.P., Szanto I. (2005). Neuronal expression of the NADPH oxidase NOX4, and its regulation in mouse experimental brain ischemia. Neuroscience.

[68] Juhasz A., Ge Y., Markel S., Chiu A., Matsumoto L., Van B.J., Roy K., Doroshow J.H. (2009). Expression of NADPH oxidase homologues and accessory genes in human cancer cell lines, tumours and adjacent normal tissues. Free Radic. Res..

[69] Si J., Fu X., Behar J., Wands J., Beer D.G., Souza R.F., Spechler S.J., Lambeth D., Cao W. (2007). NADPH oxidase NOX5-S mediates acid-induced cyclooxygenase-2 expression via activation of NF-κB in Barrett’s esophageal adenocarcinoma cells. J. Biol. Chem..

[70] Ushio-Fukai M., Nakamura Y. (2008). Reactive oxygen species and angiogenesis: NADPH oxidase as target for cancer therapy. Cancer Lett..

[71] Luxen S., Belinsky S.A., Knaus U.G. (2008). Silencing of DUOX NADPH oxidases by promoter hypermethylation in lung cancer. Cancer Res..

[72] Chen S., Ling Q., Yu K., Huang C., Li N., Zheng J., Bao S., Cheng Q., Zhu M., Chen M. (2016). Dual oxidase 1: A predictive tool for the prognosis of hepatocellular carcinoma patients. Oncol. Rep..

[73] Little A.C., Sulovari A., Danyal K., Heppner D.E., Seward D.J., van der Vliet A. (2017). Paradoxical roles of dual oxidases in cancer biology. Free Radic. Biol. Med..

[74] Chen S.S., Yu K.K., Ling Q.X., Huang C., Li N., Zheng J.M., Bao S.X., Cheng Q., Zhu M.Q., Chen M.Q. (2016). The combination of three molecular markers can be a valuable predictive tool for the prognosis of hepatocellular carcinoma patients. Sci. Rep..

[75] Wu Y., Antony S., Hewitt S.M., Jiang G., Yang S.X., Meitzler J.L., Juhasz A., Lu J., Liu H., Doroshow J.H., Roy K. (2013). Functional activity and tumor-specific expression of dual oxidase 2 in pancreatic cancer cells and human malignancies characterized with a novel monoclonal antibody. Int. J. Oncol..

[76] Qi R., Zhou Y., Li X., Guo H., Gao L., Wu L., Wang Y., Gao Q. (2016). DUOX2 expression is increased in Barrett esophagus and cancerous tissues of stomach and colon. Gastroenterol. Res. Pract..

[77] Brar S.S., Corbib Z., Kennedy T.P., Hemendinger R., Thornton L., Bommarius B., Arnold R.S., Whorton A.R., Sturrock A.B., Huecksteadt T.P. (2003). NOX5 NAD(P)H oxidase regulates growth and apoptosis in DU145 prostate cancer cells. Am. J. Physiol. Cell Physiol..

[78] Kumar B., Koul S., Khandrika L., Meacham R.B., Koul H.K. (2008). Oxidative stress in inherent in prostate cancer cells and is required for aggressive phenotype. Cancer Res..

[79] Höll M., Koziel R., Schäfer G., Pircher H., Pauck A., Hermann M., Klocker H., Jansen-Dürr P., Sampson N. (2016). ROS signaling by NADPH oxidase 5 modulates the proliferation and survival of prostate carcinoma cells. Mol. Carcinog..

[80] Arbiser J.L., Petros J., Klafter R., Govindajaran B., McLaughlin E.R., Brown L.F., Cohen C., Moses M., Kilroy S., Arnold R.S. (2001). Reactive oxygen generated by Nox1 triggers the angiogenic switch. Proc. Natl. Acad. Sci. USA.

[81] Lim S.D., Sun C.Q., Lambeth J.D., Marshall F., Amin M., Chung L., Petros J.A., Arnold R.S. (2005). Increased Nox1 and hydrogen peroxide in prostate cancer. Prostate.

[82] Deep G., Kumar R., Jain A.K., Dhar D., Panigrahi G.K., Hussain A., Agarwal C., El-Elimat T., Sica V.P., Oberlies N.H. (2016). Graviola inhibits hypoxia-induced NADPH oxidase activity in prostate cancer cells reducing their proliferation and clonogenicity. Sci. Rep..

[83] Tamura R.E., Hunger A., Fernandes D.C., Laurindo F.R., Costanzi-Strauss E., Strauss B.E. (2017). Induction of oxidants distinguishes susceptibility of prostate carcinoma cell lines to p53 gene transfer mediated by an improved adenoviral vector. Hum. Gene Ther..

[84] Arnold R.S., He J., Remo A., Ritsick D., Yin-Goen Q., Lambeth J.D., Datta M.W., Young A.N., Petros J.A. (2007). Nox1 expression determines cellular reactive oxygen and modulates c-fos-induced growth factor, interleukin-8 and cav-1. Am. J. Pahol..

[85] Pettigrew C.A., Clerkin J.S., Cotter T.G. (2012). DUOX enzyme activity promotes AKT signalling in prostate cancer cells. Anticancer Res..

[86] Tam N., Gao Y., Leung Y., Ho S.M. (2003). Androgenic regulation of oxidative stress in the rat prostate: Involvement of NAD(P)H oxidase and antioxidant defense machinery during prostatic involution and regrowth. Am. J. Pathol..

[87] Lu J.P., Monardo L., Bryskin I., Hou Z.F., Trachtenberg J., Wilson B.C., Pinthus J.H. (2010). Androgens induce oxidative stress and radiation resistance in prostate cancer cells though NADPH oxidase. Prostate Cancer Prostatic Dis..

[88] Lu J.P., Hou Z.F., Duivenvoorden W.C., Whelan K., Honig A., Pinthus J.H. (2012). Adiponectin inhibits oxidative stress in human prostate carcinoma cells. Prostate Cancer Prostatic Dis..

[89] Antony S., Wu Y., Hewitt S.M., Anver M.R., Butcher D., Jiang G., Meitzler J.L., Liu H., Juhasz A., Lu J. (2013). Characterization of NADPH oxidase 5 expression in human tumors and tumor cell lines with a novel mouse monoclonal antibody. Free Radic. Biol. Med..

[90] Huang W.C., Li X., Liu J., Lin J., Chung L.W. (2012). Activation of androgen receptor, lipogenesis, and oxidative stress converged by SREBP-1 is responsible for regulating growth and progression of prostate cancer cells. Mol. Cancer Res..

[91] Block K., Gorin Y., Hoover P., Williams P., Chelmicki T., Clark R.A., Yoneda T., Abboud H.E. (2007). NAD(P)H oxidases regulate HIF-2α protein expression. J. Biol. Chem..

[92] Gregg J.L., Turner R.M., Chang G., Joshi D., Zhan Y., Chen L., Maranchie J.K. (2014). NADPH oxidase NOX4 supports renal tumorigenesis by promoting the expression and nuclear accumulation of HIF2α. Cancer Res..

[93] Chang G., Chen L., Lin H.M., Lin Y., Maranchie J.K. (2012). Nox4 inhibition enhances the cytotoxicity of cisplatin in human renal cancer cells. J. Exp. Ther. Oncol..

[94] Shanmugasundaram K., Block K. (2016). Renal carcinogenesis, tumor heterogeneity, and reactive oxygen species: Tactics evolved. Antioxid. Redox Signal..

[95] Tsujikawa K., Koike K., Kitae K., Shinkawa A., Arima H., Suzuki T., Tsuchiya M., Makino Y., Furukawa T., Konishi N. (2007). Expression and sub-cellular localization of human ABH family molecules. J. Cell. Mol. Med..

[96] Choudhary S., Rathore K., Wang H.C. (2011). Differential induction of reactive oxygen species through Erk1/1 and Nox-1 by FK228 for selective apoptosis of oncogenic H-Ras-expressing human urinary bladder cancer J82 cells. J. Cancer Res. Clin. Oncol..

[97] Shimada K., Fujii T., Tsujikawa K., Anai S., Fujimoto K., Konishi N. (2012). ALKBH3 contributes to survival and angiogenesis of human urothelial carcinoma cells through NADPH oxidase and Tweak/Fn14/VEGF signals. Clin. Cancer Res..

[98] Shimada K., Fujii T., Anai S. (2011). ROS generation via NOX4 and its utility in the cytological diagnosis of urothelial carcinoma of the urinary bladder. BMC Cancer.

[99] Kim E.Y., Seo J.M., Kim C., Lee J.E., Lee K.M., Kim J.H. (2010). BLT2 promotes the invasion and metastasis of aggressive bladder cancer cells through a reactive oxygen species-linked pathway. Free Radic. Biol. Med..

[100] Matsuo T., Miyata Y., Asai A., Sagara Y., Furusato B., Fukuoka J., Sakai H. (2017). Green tea polyphenol induces changes in cancer-related factors in an animal model of bladder cancer. PLoS ONE.

[101] Miyata Y., Mitsunari K., Asai A., Takehara K., Mochizuki Y., Sakai H. (2015). Pathological significance and prognostic role of microvessel density, evaluated using CD31, CD34, and CD105 in prostate cancer patients after radical prostatectomy with neoadjuvant therapy. Prostate.

[102] Abid M.R., Spokes S.C., Shih W.C., Aird W.C. (2007). NAPDH oxidase activity selectively modulates vascular endothelial growth factor signaling pathway. J. Biol. Chem..

[103] Kilmova T., Chandel N.S. (2008). Mitochondrial complex III regulates hypoxic activation of HIF. Cell Death Differ..

[104] Jing Y., Liu L.Z., Jiang Y., Zhu Y., Guo N.L., Barnett J., Rojanasakul Y., Agani F., Jiang B.H. (2012). Cadmium increases HIF-1 and VEGF expression through ROS, ERK, and Akt signaling pathways and induces malignant transformation of human bronchial epithelial cells. Toxicol. Sci..

[105] Gao N., Ding M., Zheng J.Z., Zhang Z., Leonard S.S., Liu K.J., Shi X., Jiang B.H. (2002). Vanadate-induced expression of hypoxia-inducible factor 1 alpha and vascular endothelial growth factor through phosphatidylinositol 3-kinase/Akt pathway and reactive oxygen species. J. Biol. Chem..

[106] Turcotte S., Desrosiers R.R., Béliveau R. (2003). HIF-1α mRNA and protein upregulation involves Rho GTPase expression during hypoxia in renal cell carcinoma. J. Cell Sci..

[107] Shin D.H., Dier U., Melendez J.A., Hempel N. (2015). Regulation of MMP-1 expression in response to hypoxia is dependent on the intracellular redox status of metastatic bladder cancer cells. Biochim. Biophys. Acta.

[108] Coso S., Harrison I., Harrison C.B., Vinh A., Sobey C.G., Drummond G.R., Williams E.D., Selemidis S. (2012). NADPH oxidases as regulators of tumor angiogenesis: Current and emerging concepts. Antioxid. Redox Signal..

[109] Prieto-Bermejo R., Hernández-Hernández A. (2017). The Importance of NADPH Oxidases and Redox Signaling in Angiogenesis. Antioxidants (Basel).

[110] Gao N., Shen L., Zhang Z., Leonard S.S., He H., Zhang X.G., Shi X., Jiang B.H. (2004). Arsenite induces HIF-1α and VEGF through PI3K, Akt and reactive oxygen species in DU145 human prostate carcinoma cells. Mol. Cell. Biochem..

[111] Kou X., Fan J., Chen N. (2017). Potential molecular targets of ampelopsin in prevention and treatment of cancers. Anticancer Agents Med. Chem..

[112] Zhang M., Liu C., Zhang Z., Yang S., Zhang B., Yin L., Swarts S., Vidyasagar S., Zhang L., Okunieff P. (2014). A new flavonoid regulates angiogenesis and reactive oxygen species production. Adv. Exp. Med. Biol..

[113] Delle M.S., Sanità P., Calgani A., Schenone S., Botta L., Angelucci A. (2014). Src inhibition potentiates antitumoral effect of paclitaxel by blocking tumor-induced angiogenesis. Exp. Cell Res..

[114] Wu C.T., Chen M.F., Chen W.C., Hsieh C.C. (2013). The role of IL-6 in the radiation response of prostate cancer. Radiat. Oncol..

[115] Alcayaga-Miranda F., González P., Lopez-Verrilli A., Varas-Godoy M., Aguila-Diaz C., Contreras L., Khoury M. (2016). Prostate tumor-induced angiogenesis is blocked by exosomes derived from menstrual stem cells through the inhibition of reactive oxygen species. Oncotarget.

[116] Golovine K., Makhov P., Naito S., Raiyani H., Tomaszewski J., Mehrazin R., Tulin A., Kutikov A., Uzzo R.G., Kolenko V.M. (2015). Piperlongumine and its analogs down-regulate expression of c-Met in renal cell carcinoma. Cancer Biol. Ther..

[117] Li W., Liu M., Xu Y.F., Feng Y., Che J.P., Wang G.C., Zheng J.H. (2014). Combination of quercetin and hyperoside has anticancer effects on renal cancer cells through inhibition of oncogenic microRNA-27a. Oncol. Rep..

[118] Tanaka N., Miyajima A., Kosaka T., Shirotake S., Hasegawa M., Kikuchi E., Oya M. (2010). Cis-dichlorodiammineplatinum upregulates angiotensin II type 1 receptors through reactive oxygen species generation and enhances VEGF production in bladder cancer. Mol. Cancer Ther..

[119] Shirotake S., Miyajima A., Kosaka T., Tanaka N., Maeda T., Kikuchi E., Oya M. (2011). Angiotensin II type 1 receptor expression and microvessel density in human bladder cancer. Urology.

[120] Li D., Urta E., Kimura T., Yamamoto Y., Osaki T. (2004). Reactive oxygen species (ROS) control the expression of Bcl-2 family proteins by regulating their phosphorylation and ubiquitination. Cancer Sci..

[121] Scheit K., Bauer G. (2015). Direct and indirect inactivation of tumor cell protective catalase by salicylic acid and anthocyanidins reactivates intercellular ROS signaling and allows for synergistic effects. Carcinogenesis.

[122] Bauer G. (2017). Central signaling elements of intercellular reactive oxygen/nitrogen species-dependent induction of apoptosis in malignant cells. Anticancer Res..

[123] Luanpitpong S., Chanvorachote P., Stehlik C., Tse W., Callery P.S., Wang L., Rojanasakul Y. (2013). Regulation of apoptosis by Bcl-2 cysteine oxidation in human lung epithelial cells. Mol. Biol. Cell.

[124] Moloney J.N., Cotter T.G. (2017). ROS signalling in the biology of cancer. Semin. Cell Dev. Biol..

[125] Zhao Y., Hu X., Liu Y., Dong S., Wen Z., He W., Zhang S., Huang Q., Shi M. (2017). ROS signaling under metabolic stress: Cross-talk between AMPK and AKT pathway. Mol. Cancer.

[126] Ding Y., Ren K., Dong H., Song F., Chen J., Guo Y., Liu Y., Tao W., Zhang Y. (2017). Flavonoids from persimmon (*Diospyros kaki* L.) leaves inhibit proliferation and induce apoptosis in PC-3 cells by activation of oxidative stress and mitochondrial apoptosis. Chem. Biol. Interact..

[127] Elkady A.I. (2017). Anethole inhibits the proliferation of human prostate cancer cells via induction of cell cycle arrest and apoptosis. Anticancer Agents Med. Chem..

[128] Deng Y., Li Y., Yang F., Zeng A., Yang S., Luo Y., Zhang Y., Xie Y., Ye T., Xia Y. (2017). The extract from Punica granatum (pomegranate) peel induces apoptosis and impairs metastasis in prostate cancer cells. Biomed. Pharmacother..

[129] Ahmad M., Sahabjada, Akhtar J., Hussain A., Badaruddeen, Arshad M., Mishra A. (2017). Development of a new rutin nanoemulsion and its application on prostate carcinoma PC3 cell line. EXCLI J..

[130] Lin J.F., Tsai T.F., Yang S.C., Lin Y.C., Chen H.E., Chou K.Y., Hwang T.I. (2017). Benzyl isothiocyanate induces reactive oxygen species-initiated autophagy and apoptosis in human prostate cancer cells. Oncotarget.

[131] Rodriguez-Garcia A., Hevia D., Mayo J.C., Gonzalez-Menendez P., Coppo L., Lu J., Holmgren A., Sainz R.M. (2017). Thioredoxin 1 modulates apoptosis induced by bioactive compounds in prostate cancer cells. Redox Biol..

[132] Zhu W.B., Tian F.J., Liu L.Q. (2017). Chikusetsu (CHI) triggers mitochondria-regulated apoptosis in human prostate cancer via reactive oxygen species (ROS) production. Biomed. Pharmacother..

[133] Ryu S., Lim W., Bazer F.W., Song G. (2017). Chrysin induces death of prostate cancer cells by inducing ROS and ER stress. J. Cell. Physiol..

[134] Kim K.Y., Kim S.H., Yu S.N., Park S.G., Kim Y.W., Nam H.W., An H.H., Yu H.S., Kim Y.W., Ji J.H. (2017). Lasalocid induces cytotoxic apoptosis and cytoprotective autophagy through reactive oxygen species in human prostate cancer PC-3 cells. Biomed. Pharmacother..

[135] Lim W., Park S., Bazer F.W., Song G. (2017). Naringenin-induced apoptotic cell death in prostate cancer cells Is mediated via the PI3K/AKT and MAPK signaling pathways. J. Cell. Biochem..

[136] Lim W., Jeong M., Bazer F.W., Song G. (2017). Coumestrol inhibits proliferation and migration of prostate cancer cells by regulating AKT, ERK1/2, and JNK MAPK cell signaling cascades. J. Cell. Physiol..

[137] Chen M., Zhou B., Zhong P., Rajamanickam V., Dai X., Karvannan K., Zhou H., Zhang X., Liang G. (2017). Increased intracellular reactive oxygen species mediates the anti-cancer effects of WZ35 via activating mitochondrial apoptosis pathway in prostate cancer cells. Prostate.

[138] Kim K.Y., Park K.I., Kim S.H., Yu S.N., Park S.G., Kim Y.W., Seo Y.K., Ma J.Y., Ahn S.C. (2017). Inhibition of autophagy promotes salinomycin-induced apoptosis via reactive oxygen species-mediated PI3K/AKT/mTOR and ERK/p38 MAPK-dependent signaling in human prostate cancer. Int. J. Mol. Sci..

[139] Park S.G., Kim S.H., Kim K.Y., Yu S.N., Choi H.D., Kim Y.W., Nam H.W., Seo Y.K., Ahn S.C. (2017). Toyocamycin induces apoptosis via the crosstalk between reactive oxygen species and p38/ERK MAPKs signaling pathway in human prostate cancer PC-3 cells. Pharmacol. Rep..

[140] Qiu M., Chen L., Tan G., Ke L., Zhang S., Chen H., Liu J. (2017). JS-K promotes apoptosis by inducing ROS production in human prostate cancer cells. Oncol. Lett..

[141] Thamilselvan V., Menon M., Stein G.S., Valeriote F., Thamilselvan S. (2017). Combination of carmustine and selenite inhibits EGFR mediated growth signaling in androgen-independent prostate cancer cells. J. Cell. Biochem..

[142] Thamilselvan V., Menon M., Thamilselvan S. (2016). Combination of carmustine and selenite effectively inhibits tumor growth by targeting androgen receptor, androgen receptor-variants, and Akt in preclinical models: New hope for patients with castration resistant prostate cancer. Int. J. Cancer.

[143] Wright C., Iyer A.K.V., Kaushik V., Azad N. (2017). Anti-tumorigenic potential of a novel orlistat-AICAR combination in prostate cancer cells. J. Cell. Biochem..

[144] Cao J., Wang H., Chen F., Fang J., Xu A., Xi W., Zhang S., Wu G., Wang Z. (2016). Galangin inhibits cell invasion by suppressing the epithelial-mesenchymal transition and inducing apoptosis in renal cell carcinoma. Mol. Med. Rep..

[145] Zhong W.F., Wang X.H., Pan B., Li F., Kuang L., Su Z.X. (2016). Eupatilin induces human renal cancer cell apoptosis via ROS-mediated MAPK and PI3K/AKT signaling pathways. Oncol. Lett..

[146] Park J.E., Park B., Chae I.G., Kim D.H., Kundu J., Kundu J.K., Chun K.S. (2016). Carnosic acid induces apoptosis through inactivation of Src/STAT3 signaling pathway in human renal carcinoma Caki cells. Oncol. Rep..

[147] Wu J., Zheng W., Rong L., Xing Y., Hu D. (2017). Bicyclol exerts an anti-tumor effect via ROS-mediated endoplasmic reticulum stress in human renal cell carcinoma cells. Biomed. Pharmacother..

[148] Zhao J., He Q., Gong Z., Chen S., Cui L. (2016). Niclosamide suppresses renal cell carcinoma by inhibiting Wnt/β-catenin and inducing mitochondrial dysfunctions. Springerplus.

[149] Yin P., Jia J., Li J., Song Y., Zhang Y., Chen F. (2016). ABT-737, a Bcl-2 selective inhibitor, and chloroquine synergistically kill renal cancer cells. Oncol. Res..

[150] Gillissen B., Richter A., Richter A., Preissner R., Schulze-Osthoff K., Essmann F., Daniel P.T. (2017). Bax/Bak-independent mitochondrial depolarization and reactive oxygen species induction by sorafenib overcome resistance to apoptosis in renal cell carcinoma. J. Biol. Chem..

[151] Wang L., Azad N., Kongkaneramit L., Chen F., Lu Y., Jiang B.H., Rojanasakul Y. (2008). The Fas death signaling pathway connecting reactive oxygen species generation and FLICE inhibitory protein down-regulation. J. Immunol..

[152] Lee S.J., Noh H.J., Sung E.G., Song I.H., Kim J.Y., Kwon T.K., Lee T.J. (2011). Berberine sensitizes TRAIL-induced apoptosis through proteasome-mediated downregulation of c-FLIP and Mcl-1 proteins. Int. J. Oncol..

[153] Han M.A., Woo S.M., Min K.J., Kim S., Park J.W., Kim D.E., Kim S.H., Choi Y.H., Kwon T.K. (2015). 6-Shogaol enhances renal carcinoma Caki cells to TRAIL-induced apoptosis through reactive oxygen species-mediated cytochrome c release and down-regulation of c-FLIP(L) expression. Chem. Biol. Interact..

[154] Park E.J., Chauhan A.K., Min K.J., Park D.C., Kwon T.K. (2016). Thymoquinone induces apoptosis through downregulation of c-FLIP and Bcl-2 in renal carcinoma Caki cells. Oncol. Rep..

[155] Lu C.C., Shen C.H., Chang C.B., Hsieh H.Y., Wu J.D., Tseng L.H., Hwang D.W., Chen S.Y., Wu S.F., Chan M.W. (2017). Guizhi Fuling Wan as a novel agent for intravesical treatment for bladder cancer in mouse model. Mol. Med..

[156] Romanov V., Whyard T.C., Waltzer W.C., Grollman A.P., Rosenquist T. (2015). Aristolochic acid-induced apoptosis and G2 cell cycle arrest depends on ROS generation and MAP kinases activation. Arch. Toxicol..

[157] Li Y., Wen J.M., Du C.J., Hu S.M., Chen J.X., Zhang S.G., Zhang N., Gao F., Li S.J., Mao X.W. (2017). Thymol inhibits bladder cancer cell proliferation via inducing cell cycle arrest and apoptosis. Biochem. Biophys. Res. Commun..

[158] Qiu M., Chen L., Tan G., Ke L., Zhang S., Chen H., Liu J. (2015). A reactive oxygen species activation mechanism contributes to JS-K-induced apoptosis in human bladder cancer cells. Sci. Rep..

[159] Yu G.Q., Dou Z.L., Jia Z.H. (2017). 5-bromo-3-(3-hydroxyprop-1-ynyl)-2*H*-pyran-2-one induces apoptosis in T24 human bladder cancer cells through mitochondria-dependent signaling pathways. Mol. Med. Rep..

[160] Saleem A., Dvorzhinski D., Santanam U., Mathew R., Bray K., Stein M., White E., DiPaola R.S. (2012). Effect of dual inhibition of apoptosis and autophagy in prostate cancer. Prostate.

[161] Hsin I.L., Wang S.C., Li J.R., Ciou T.C., Wu C.H., Wu H.M., Ko J.L. (2016). Immunomodulatory proteins FIP-gts and chloroquine induce caspase-independent cell death via autophagy for resensitizing cisplatin-resistant urothelial cancer cells. Phytomedicine.

